# Clinical characteristics of adolescents and adults with Sickle Cell Disease and barriers to transition to adult care at Mulago Hospital, Uganda: A mixed methods study

**DOI:** 10.21203/rs.3.rs-6195381/v1

**Published:** 2025-03-17

**Authors:** Racheal Owomuhangi, Charles Karamagi, Grace Ndeezi, Japheth Kwiringira, Deogratias Munube, Sarah Kiguli, Robert Opika Opoka, Ruth Namazzi

**Affiliations:** Makerere University; Makerere University; Makerere University; Kyambogo University; Makerere University; Makerere University; Makerere University; Makerere University

**Keywords:** Transition, sickle cell disease, Paediatrics, young adults

## Abstract

**Background:**

Outcome of patients with sickle cell disease (SCD) has improved greatly over the past 60 years with several studies showing improved survival into adulthood due to advancement in medical care. A successful transition is critical for optimal health outcomes. However, health care delivery systems that support the optimal transfer from pediatric to adult care have not kept pace with the growing adult population. Mulago pediatric sickle cell clinic has faced multiple challenges with transition to adult care that are not well documented. The objective of this study was to describe the clinical characteristics of adolescents and adults with sickle cell disease and barriers to adult care at Mulago hospital.

**Methods:**

This was a mixed methods cross sectional study with both qualitative and quantitative data collection methods conducted among patients attending the pediatric sickle cell clinic at Mulago hospital, their caregivers and health care workers. A registry and medical records review was done to obtain data for the quantitative arm. The qualitative component consisted of 30 in-depth-interviews involving patients and care givers and 10 key informant interviews with healthcare workers. Quantitative data was coded and entered into Epidata version 4.6 and then exported to STATA 14 for analysis. Qualitative data was analyzed using the content thematic approach.

**RESULTS:**

The proportion of patients aged 14 years and above still attending the pediatric clinic was 21.6%. Barriers to transition of care as expressed by care givers and patients were limited knowledge on transition, attachment to their pediatric careers and negative experiences in the adult clinics. Health care system barriers included poorly organized adult clinics with few working days compared to the paediatric clinic that operates daily. This was compounded by lack of policies and guidelines on transition, inadequate human resource and limited access to the essential drugs in the adult clinics.

**Conclusions and recommendations:**

There is still a large proportion of adults and young adults (AYAs) still attending the pediatric sickle cell clinic and barriers to transition were not only sociodemographic but also psychosocial and health system related. There is need for better planning and preparation with better patient centered interventions in order to improve transition.

## Background

Sickle cell disease (SCD) is a chronic inherited illness that occurs primarily in individuals of sub–Saharan African and southwest Asian ancestry who are homozygous for the gene controlling hemoglobin. Clinically, erythrocyte abnormalities manifest as hemolytic anemia and the inability of sickled cells to pass through the micro vasculature leading to vaso-occlusion and end-organ ischemia-reperfusion injury and infarction(1).

Currently, the prevalence of sickle cell anemia worldwide is 4.4 million people and 43 million people with sickle cell trait with 75% of these sickle cell cases being in Africa(2, 3). The overall prevalence of sickle cell trait in Uganda is 13.3% and that of sickle cell anaemia is 0.8%, according to the Uganda sickle cell surveillance study with the highest burden being in east central region(4).

Survival of patients with sickle cell disease has greatly improved over the past 60 years. From being described as the disease of childhood in 1960 to about 85% of hemoglobin SS (HbSS) patients living to adult hood 25 years later(5). This improvement in survival is attributed to enhanced pediatric care such as neonatal screening, immunizations, penicillin prophylaxis and disease modifying drugs like hydroxyurea(5–7).

Given the medical advances that have transformed SCD into a lifelong condition, transition of young adults with sickle cell disease from pediatric to adult medical care is an important priority in order provide quality care. Transition is particularly critical because adolescence is associated with a number of complications, risky behaviors and greater vulnerability for other disorders among other significant life changes in emerging adulthood. Transition is defined as the multifarious, vibrant, purposeful planned movement of young adults with chronic physical and medical conditions from child centered to adult-oriented health care systems(1).

However, transition from pediatric to adult care services has encountered multiple problems globally such as lack of important information about SCD and transition, psychosocial factors, poor communication and follow-up between pediatric and adult providers, lack of trust in the adult care providers as well as poor adaptation to adult life(8). Without transition, maximizing lifelong functioning and delivery of high quality, developmentally suitable health care services that carry the patient into adult care is difficult. Hence adolescents resort to managing their disease complications at home, with consequent loss to follow up leading to accumulation of co-morbidities and increased mortality and morbidity(8). In order to avoid excess morbidity and mortality country programs should conduct studies to address the challenges associated with transition from paediatric to adult care to guide the best practices. (9).

This study describes the proportion of patients aged 14 years and above still attending the paediatric SCC and barriers to transition from the pediatric to the adult clinic at the largest sickle cell disease clinic in Uganda

## Methods

### Study design.

This was a mixed methods cross sectional study with both qualitative and quantitative components. The quantitative part of the study was retrospective in nature and used data of the year 2019 to determine the proportion of patients aged 14 years and above that attended the Mulago hospital sickle cell clinic in that year and described their clinical and socio-demographic characteristics. The qualitative component of the study was carried out to explore the barriers to transition of patients from pediatric to adult care.

## Study setting

The study was conducted at the Mulago National Hospital Sickle Cell Clinic (MHSCC), Kampala, Uganda. Mulago Hospital is Uganda’s National Referral and teaching hospital for Makerere College of Health Sciences, Kampala. It is located on the upper Northwest of Mulago hill. It receives patients referred from health facilities within and outside Kampala District. The Department of Pediatrics and Child Health runs 6 specialized clinics, of which is the Sickle Cell Clinic. The MHSCC has 15,500 registered patients with about 800–900 client clinic visits per month and an average of 70 to 80 patients per day, attending either for sick visits or routine follow up. The clinic is run by pediatric haematologists, hematology fellows, medical officers, clinical officers, senior house officers, nurses, pharmacy technicians and a few laboratory personnel. There is always about 8–12 health care workers at each clinic visit to render the required services.

Standard care includes: Penicillin V prophylaxis for all under-fives, Analgesia, Oral Rehydration salts (ORS) for hydration, daily Folic acid for anemia prevention and monthly Fansidar ^®^ for Malaria chemo-prophylaxis. Hydroxyurea is provided for free on a limited scale at the MHSCC depending on available stocks. At each visit, the attending health care worker does a thorough clinical evaluation including history and physical examination, requests for laboratory investigations that include a blood smear for malaria parasites and a complete blood count. Depending on the clinician’s assessment, and where required the laboratory parameters and the MHSCC protocols, the patient is managed as either an outpatient, in the day-care unit or hospitalized to Acute Care Unit (the pediatric emergency ward) for cases that require more monitoring and treatment.

The patients who are aged 15 years and above are given a verbal communication to seek care at the adult clinic in Kiruddu hospital or the new Mulago unit at the 4th floor hematology clinic. Kiruddu National referral hospital is currently an independent National Referral Hospital Kampala city council based. It is located on Buziga Hill, Makindye Division in Kampala. It’s approximately 13 kilometres southeast of Mulago Hospital. The internal medicine department in Kiruddu runs many clinics including the hematology clinic that runs every Thursday 8am-5pm.

## Study participants

For the quantitative arm, a sample of 384 medical files of patients with confirmed and documented evidence (HB electrophoresis) of sickle cell disease aged 14 years and above that attended Mulago pediatric sickle cell clinic from January to December 2019 were selected using probability proportional to size to avoid biased sampling of patients who came early during the year. The year 2019 was used because this was a COVID-19 pre-pandemic year giving us true representation of clinic attendance. The sample size was obtained Using Kish Leslie formula for cross-sectional studies with the assumption of a 50% transition rate based on a study by Mariam kayle at al on transition to adult care in sickle cell disease (10).

For the qualitative arm, all patients with sickle cell disease aged 14 years and above attending the pediatric sickle cell clinic of Mulago National Referral Hospital, their caregivers and health care service providers at MHSCC who had given consent and assent, within the study period (May- July 2022) were included in the study. Patients who were critically ill and in need of urgent emergency care were excluded.

Participants of the qualitative arm were purposively selected for the interviews.

## Study procedure:

### For quantitative data collection:

A desk review of the registry was done by the principal investigator to obtain the number of patients aged 14 years and above attending the MHSCC. Upon which the proportion of patients aged 14 years and above was calculated. Then a total of 384 medical file records of patients with sickle cell disease aged 14 years and above who attended clinic from January to December 2019 were retrieved from the records office. Assuming 50% of patients were above 14 years of age, using and Leslie formula. the sample size used was 384 patients. The socio-demographic and clinical characteristics of the participants that were routinely recorded in the medical files were extracted using a pretested data extraction tool.

This data was collected concurrently with the qualitative data since one was a medical records review and the other was physical interviews of the participants attending the clinic during the study period.

### For qualitative data collection

Key informant interviews and in-depth interviews were used to obtain an in-depth understanding of the barriers to transition. Patients aged 14 years and above with SCD and their care givers were identified and consented for the study if they fulfilled the inclusion criteria. Inclusion criteria was as follows:

- Patients above 18 years who gave consent in addition to assent for those aged 14 up to 17 years.
- Primary Caregivers of sickle cell patients aged 14 years and above attending MHSCC who had given consent.
- Health care providers (Head of the sickle cell clinic, hematology oncology fellows, senior house officers, medical officers, nurses and pharmacy technicians) at MHSCC who interact with these patients that were present during the study period (May- July 2022) and gave consent.

Interviews were conducted in a private area that had been designated as our study space by the principal investigator assisted by a study doctor (research assistant) and one of the investigators who was well versed with qualitative research methods. Assessment for the routine or sick visit including history taking, physical examination and drug refills were done after completing the study interviews. The interviews were carried out every Monday, Wednesday and Friday aside from public holidays falling on week days until the sample size was accrued. Each participant was assigned a study number which was appended on the patient’s file, visitation card and exercise book in addition to colour coding to avoid double enrolment.

### In-depth interviews

In-depth interviews were conducted among patients aged 14 years and above attending MHSCC and their accompanying care givers using a semi-structured interview guide to explore the barriers to transition at MHSCC. We opted for In-depth interviews because they provided free sharing space, confidentiality and privacy for the adolescents to freely express their views. Thirty in-depth interviews were conducted and were adequate to ascertain a clear pattern. Participants were selected putting into consideration the age and gender in order to ensure that each category was well represented. We had 10 in-depth interviews with patients aged 14–18 years and another 10 with those aged 18 years and above attending MHSCC stratified by gender (half were males and the other half females). Ten accompanying care givers of the adolescents between ages 14–18 years were also interviewed. We were unable to interview caregivers of patients above 18 years because almost all of them attended the clinic by themselves without an escorting caregiver. Interviews between patients and care givers were carried out separately in order to provide enough room for the adolescents and young adults to express their views without worry about their caregivers. Discussions lasted at least 30–45 minutes. The interviews were held in a neutral room that allows privacy. One of the investigators who is well grounded in qualitative research methodology guided the session. All interviews were in the language best spoken by the participant which was mostly Luganda and English. Interviews we then tape recorded in order not to miss any content, if participants were not comfortable with the recording, then instead notes were taken.

### Key informant interviews

A semi structured key informant interview guide was used by a trained research assistant and the investigators to collect information on the current practices on transitioning, and health provider’s perceptions towards transition of care as well as the barriers. The key informants were approached or called on phone to schedule the convenient time for interviews by the principal investigator. Once the appropriate time was confirmed then each key informant was approached according to the schedule in areas of their own convenience. Ten Interviews were conducted and each averaged 30–40 minutes. All of these interviews were conducted in english The key informants included the head of the sickle cell clinic, two pediatric hematologists, two hematology oncology fellows, two senior house officers, two nurses and one pharmacy technician.

### Statistical analysis

Quantitative data was coded and double entered into Epidata version 4.6 and then exported to STATA version 14.0 for analysis. The proportion of patients above 14 years attending the Mulago Hospital sickle cell clinic was analysed as a frequency distribution and other categorical variables was presented in form of frequencies and percentages.

Qualitative data from in-depth interviews and key informant interviews was analysed using content thematic approach. Audio recordings from in-depth interviews and key informant interviews were transcribed through repeated careful listening and generally written verbatim by the transcriber and principal investigator and then translated in text, into English before analyzing. The principal investigator identified major themes and subthemes through open coding that was done on all transcribed interviews. Development of a list of data-driven and structural codes necessitated repeated examination of the raw data. The codes were then reviewed and revised before they are grouped into categories. To ensure that they were established through reliability, multi-step “sense making” endeavor was used. Direct quotations were used in the presentation of results.

## Results

This was a mixed methods study carried out from May 2022 to August 2022 at the pediatric sickle cell clinic of Mulago National Referral Hospital.

By 31^st^ December 2019, a total of 15515 patients were registered in the pediatric sickle cell clinic. However only 4372 patients actively attended the pediatric sickle cell clinic that year (attended clinic at least twice). Of the active patients, 955 patients (21.6%) were aged 14 years a

### Sociodemographic characteristics of SCD patients who attended the clinic in 2019

Socio-demographic and clinical characteristics of 384 files of patients selected for the study are described. Majority of the patients, 59.4%(228/384) were aged between 14 to 18 years. There were slightly more female 53.4% (205/384) than male patients. Most of the patients (53.3%) lived within 5 kilometers from Mulago hospital. The primary caregivers were in most cases a parent (66.6%) followed by 22.5%(86/384) who were responsible for their own care. **The results are summarized in table 1 in the appendix.**

### Clinical and laboratory characteristics of SCD patients above 14 years of age in 2019.

Clinical characteristics included hemoglobin type, SCD complications, weight, sexual development, clinic utilization and prescribed treatments. Of 384 SCD patients who attended the sickle cell clinic in 2019, 98.2% (377/384) had HbSS. About 9.2% (35/384) had disease complications registered in their medical files and 3.4% had comorbidities. Of the 384 patients, 288(75.4%) attended the clinic at least 4 times of which 64% (206/384 were sick visits. About 33.9% below the 5^th^ percentile weight for age score on the CDC weight for age charts. About 48.8% of patients received hydroxyurea. **Results are summarized in the table 2 in the appendix.**

### Barriers to transition of care from pediatric to adult care of SCD patients attending pediatric sickle cell clinic, Mulago Hospital

#### Health system related barriers:

These were sub-divided into barriers at the pediatric sickle cell clinic and barriers at the place where the patients were to be transitioned to (adult clinic: either hematology clinic in New Mulago 4^th^ floor or Kiruddu national referral hospital). We linked these barriers and structured them around the WHO framework that describes health systems in terms of six core components or “building blocks”: (i) Good service delivery, (ii) health workforce, (iii) health information systems, (iv) access to essential medicines, vaccines and technologies (v) financing, and (vi) leadership/governance

##### Leadership and Governance

###### Lack of transition policy

The most frequently discussed barrier by the health care providers at the sickle cell clinic was the lack of a well written protocol to better facilitate a seamless transition from pediatric to adult health care. Health workers have tried to give verbal communication for transfer which has not always been effective. For instance, one health provider said:

“So right now, there no developed guidelines yet, only that it was just unwritten understanding that we will try to transit these patients but structurally it has not been well designed, there no guiding policies thathave been put in place” (KI 009)

However, some health workers also reported limited understanding on what the transition process should entail. This affected the policy making process but also the knowledge that they passed on to the patients about transition. For instance, one key informant said:

“From your explanation of what transition is I realise now that our understanding of transition as clinicians is vague, I personally thought it means move, what have we been telling patients, my God!” (K007)

##### Access to essential medicines

###### Limited access to essential drugs and drug stock outs:

Health care providers noted limited access to essential drugs and frequent drug stock outs in the adult clinic. They also received feedback from some of the patients that had attended the adult clinics about the drug shortages especially hydroxyurea, which is an essential disease modifying drug needed to improve quality of life and morphine that is largely needed for the frequently occurring pain crises

“So, the other challenge is that medicines or drugs sometimes are not as available as they are in our clinic” (KI 009)

##### Service delivery.

###### Few clinic working days.

Health care providers reported disparities in care delivery at the adult clinic such as the inconvenient clinic days because the clinic works only once a week on Thursdays and yet the nature of sickle cell disease, patients fall sick any time. As one health care provider mentioned:

“Now for the adult clinic it runs once week in Kiruddu hospital and lower Mulago also once a week, we also have another adult clinic which runs at Uganda cancer institute also once a week so you find that the people will go but if on a day when they are not well and they find it is a day for us they will always come back to us” (KI 009)

###### Poorly structured Clinic environment

The adult clinic at New Mulago 4^th^ floor and Kiruddu hospital are not well structured. They lack an emergency unit for resuscitation of the very sick patients, there is no clinic specific laboratory or pharmacy. Patients have to walk around the blocks to access medication or even laboratory services even when in crisis. A key informant said:

“Yes, like I said the sickle cell clinic here is well structured, organized with a day care for emergencies and area of arrival, waiting shed, the clinical room, laboratory, pharmacy, but the adult clinic is not as well-structured you go to fourth floor and you see the doctor, the doctor sends you to the general hospital lab as opposed to the specific clinic lab” (KI 008)

###### Clinic is not sickle cell specific

The adult clinics both in Kiruddu and New Mulago are not sickle cell specific. The clinic reviews everyone with hematological complications which is not convenient for SCD patients. Health worker stated

“You know it’s just a hematology clinic it’s not sickle cell” it’s all hematology cases on that Thursday which is not convenient for the sicklers. So, if they would open up a specific sickle cell clinic for adults, I think they would move that side” (KI 001)

##### Health care work force

###### Inadequate health care work force

In this study participants reported another barrier of the inadequate human resource, fair and efficient to achieve the transition process. The number of health care workers in the pediatric care working at a point in time were few to take the time to explain transition to the patients. There are very few adult carers available in adult clinics to continue the chronic care. As health care workers mentioned.

“And then there are very few personnel/ staffs in the adult clinic at new Mulago and even Kiruddu only one specialist down there it’s largely the low cadre staff but here there many specialists to attend to them” (KI 008)

###### Poor coordination between the pediatric and adult health care providers

Health care providers expressed their grievances about the poor coordination between the pediatric and existing adult carers and the unwelcoming/poor attitude of the adult carers to work and patients. As a key informant stated:

“We lack cooperation with those physicians, me I don’t personally know any adult carer I can call to refer a patient to. They are never even available” (KI 007)

###### Individual barriers stated by patients and caregivers:

The major barriers to transition of care of SCD patients were divided into both care giver and patient factors. They reflected major concerns such as limited knowledge on transition, attachment to their pediatric carers, long distance to the adult care unit, negative experiences as shared by the patients and inappropriate age of transition by both parties.

##### Limited knowledge on the transition process.

Both caregivers and patients in this study expressed that their knowledge about the transition to adult health care services were limited and often absent which was more emphasized among the patients. Those that had some knowledge on the transition which was vaguely given by some health care givers did not know where the adult clinic was found and which days the clinic runs. For instance one adolescent said:

“No, I have spent 7 years attending this clinic but I have never heard about the adult clinic and nobody has ever told me. May be when I turn 18 years there is where they said you have to sign in the file. so, I only know about that, as far as transferring from pediatric care to adult care givers I haven’t heard about it” (adolescent 002,

Similary when the caregivers were asked about their knowledge of the transition process, they simply expressed:

“I have never heard about transition though sometimes he comes alone but he would still have informed me so I have never heard about it, I have never heard that even there is another clinic apart from this one” (Caregiver 008)

##### Negative Experiences

Most of the adults above 18 years of age that had experienced some adult care reported bad experiences such as the unfriendly nature of the adult carers and substandard care and most of them did not want to move back, while some adolescents 14–18years and care givers had their own negative beliefs about the differences between pediatric and adult care from the stories they had heard from patients in adult care units. Some of these stories were re-echoed by some key informants whom some of the patients had confided in. For instance, one patient expressed:

“At Kiruddu, they don’t care about people, I went there when I was in pain and they did not work on me, I lined up the whole day” (adult 004)

Caregivers and key informants also expressed similar concerns. When interviewed they said:

“Health workers in the adult clinic don’t give us much attention and yet our children are in a lot of pain” (Caregiver 006)

##### Attachment to their pediatric care givers

Concerns related to leaving their pediatric providers and the peditaric environment for a new adult provider were strongly expressed. Thoughts of terminanting relationships with their pediatric carers triggered anxiety and worry for the patients and their families. These thoughts were also mirrored by their health care providers with whom patients had shared their concerns with. The patients and pediatric providers stated:

“You really feel at home when you come at the sickle cell clinic in Mulago. I have been at Nsambya they give you the medication but then they don’t give you that touch. There is something about thesepeople it’s like they know us and they understand us, I really appreciate them. I even feel anxious just thinking about moving” (Adult 008)

## DISCUSSION

A significant proportion of adolescents aged 14 years and above and adults with sickle cell clinic still attend the paediatric care services. Ensuring effective transition from pediatric to adult care right now is a country wide priority for optimizing AYAs health and critical for prevention of mortality and, morbidity that comes with SCD.

From this study we noted a significant number of AYAS still attending pediatric clinics. One in every four patients attending the sickle cell clinic at Mulago hospital are 14 years of age and above. The current practice in transition has not been effective as seen from this study. This study was able to highlight that barriers to transition were not only psychosocial and health system related as projected from the interviews but also sociodemographic and clinical through the description of the patient characteristics.

Majority of the patients, lived within a 5-kilometer radius of the hospital. A five kilometer distance was used because it’s the recommended definition to accessible health care in terms of distance by the Universal health care of Uganda(11). This could be one of the reasons patients are still attending paediatric care because of closeness to the clinic. This was reflected in some of the in-depth interviews by the caregivers and patients that pointed out the long distance to adult clinic as one of the barriers to transfer to adult care. Our findings are similar to a study done by Mariam Kayle et al in 2019 describing the clinical characteristics of adolescents and young adult (AYAs) with SCD during transition years where 25% of AYAs lived closer to the pediatric clinic. The study emphasized that AYAs that lived close to the adult clinic were more likely to transfer to adult care than those that lived far from it (10).

Most of the Patients in our study had delayed sexual development, low weight for age. Because of their low body weight and delayed sexual maturity, they are more likely to identify as children rather than adults and hence opt to stay in pediatric care units. This was also reflected in the interviews conducted among patients and caregivers, who emphasized that their children looked young and were not fully developed for adult care. Our study reported similar findings to previous studies that have reported poor growth and delayed maturation such as a study by Babette et al that assessed effects of delayed pubertal development in SCD patients 14–29 years. The study found that 84% of subjects declined in BMI and 38% fell below 5th percentile, while puberty development was delayed by 1–2 years(12). Manuela et al also reported significant finding of lower weights and delayed sexual development in subjects with SCD than their matched controls in a study done to assess physical growth and sexual maturation in SCD patients in 2000(13).

The pediatric sickle cell clinic at Mulago is able to supply hydroxyurea without cost implications to the patient and yet the adult clinic frequently has stock-outs and medication is obtained at a fee. Presence of hydroxyurea in the clinic keeps most AYAs attending pediatric clinics since its availability in the adult clinic is unpredictable. This was reflected in the interviews that were conducted among young adults who had accessed some adult care, reporting its unavailability in adult care, making them come back to pediatric care, where they could be availed even the little doses that were present. These findings were similar to a study by Morey et al that assessed age related treatment patterns in SCD patients and found that use of hydroxyurea in adult clinics facilitated movement to adult clinics where it was available(14).

Overall, our pediatric health care providers discussed multiple themes related to barriers to transition to adult care with the most emphasized being the current format of transition has not helped the process because the clinic did not have a well written protocol to better facilitate a seamless transition from pediatric to adult health care. Pediatric providers also cited challenges in the adult care service delivery that included few clinic days and the poor arrangement in the adult clinics, unavailability of essential drugs and stock outs as well as inadequate human resource and poor coordination between the pediatric and adult carers According to their views the adult clinics worked only one day of the week, there were not specialized for only sickle cell disease patients but rather catered for all hematology cases and the clinics are not well structured. The emergency departments, laboratories and pharmacies were in a different area away from the clinic which was not convenient for patients especially those in crisis that had to move around to access the different modes of care. Extrapolating data mainly from East African studies on barriers to successful transition in HIV infected adolescents, Mbalinda et al highlighted transition barriers such as inadequate staff and poor communication between pediatric and adult care givers and attachment of the adolescents to their long-term carers(15). Jeffrey D Lebensurger et al highlighted a keep barrier to transition as the lack of communication between paediatric and adult care physicians during transition years, and poor service delivery in adult clinics(16). Wendy et al also endorsed the disjuncture relationship between the pediatric and adult cares leading to less successful transitions(17).

Patients and their caregivers in our study reflected major concerns such as limited knowledge on transition, attachment to their pediatric carers, long distance to the adult care unit, negative experiences and inappropriate age of transition.

Several young adults who had accessed adult care at a certain point in their life reported very bad experiences in adult care. The adult carers did not treat them with respect and kindness, they waited for long hours to be seen and some were not even seen at all. Some had lost their colleagues and friends to what they termed as carelessness and untimely service delivery

Thoughts of terminating relationships with their pediatric carers triggered anxiety and stress for the patients and their care givers as some of the providers had become family. Participants could not imagine replacing what they knew as the best supportive health system they had experienced for the inferior, unpleasant adult care they had heard about.

The study by Dimitropolous et al that investigated the experiences of young people who had transferred from pediatric to adult care highlighted similar experiences. It suggested that management in adult clinics was wanting with lengthy waiting hours and continuously repeating of medical information to uninterested health care givers and this led to disinterest in adult care by the patients with many returning to pediatric care or lost to follow up(18, 19).

### Strength of the study

This study utilized both quantitative and qualitative methods of data collection which offered an extensive opportunity to describe the barriers to appropriate transition of care of SCD patients at Mulago hospital. We were able to capture the views of different stakeholders in the transition process including the patients themselves, caregivers and their health workers. This gave us a broad view of what the problem was.

The study was carried out at the Mulago pediatric sickle cell clinic which is a tertiary facility for sickle cell management in the country. Therefore, the results are indicative of what takes place in the public health facilities in Uganda with regards to transitioning patients with chronic illnesses to adult care.

### Study limitations

This being a retrospective study which involved review of patient records, it was not possible to verify information with providers or patients in real time to correct inconsistencies. However, where necessary, a phone call was made to the patient or next of kin to verify the inconsistency. Additionally, missing documentation of information such as symptoms (Vaso-occlusive episodes), measurements such as height, complications of disease and comorbidities were considered as absent and yet could have been present. This could have potentially led to misinterpretation of patient characteristics.

In this study, data was limited to only one health care system (pediatric care). We were unable to assess the views of the adult health carers on the barriers to transition of care and since most of the views by the key informants on the health care system barriers seemed to be failures in adult care system, interviewing these health workers would have helped triangulate results.

## Conclusion

Our study demonstrated a significant number of AYAs still attending the Pediatrics clinics.

It also pointed out that our current practice on transition is not ideal and standard for chronic care management with multiple barriers such as lack of policy, patient and care giver limited knowledge on transition, lack of proper well-organized infrastructure for adult care, inadequate human resources and drug stock outs among many

There is an urgent need to address the barriers in order to achieve a seamless transition process.

## Recommendation

Based on the information gathered from this study, it’s important to recognize and acknowledge that the “one size fits all” approach to transition may not be appropriate for this setting, significant changes and strategies should be developed in collaboration with patients, care givers as well as the adult health care professionals to better prepare for this process.

## Figures and Tables

**Figure 1:**
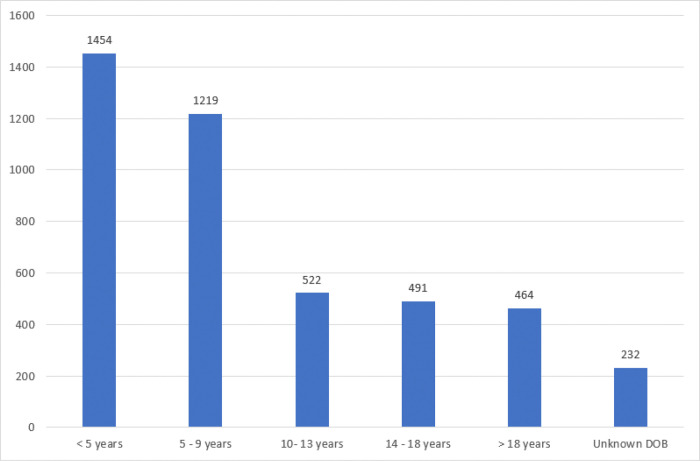
Figure 3: Bar graph showing the total number of patients distributed by age that actively attended the pediatric sickle clinic, Mulago Hospital in the year 2019.

## Data Availability

The original data set generated and/or analyzed during the study will be made available by the corresponding author upon request.

## Abbreviations

ACU: Acute Care Unit
AYAs: Adolescents and Young Adults
HbSS: Homozygous for the Sickle Cell gene
KNRH: Kiruddu National Referral Hospital
MNRH: Mulago National Referral Hospital
MHSCC: Mulago Hospital Sickle Cell Clinic
SCA: Sickle Cell Anemia
SCC: Sickle Cell clinic
SCD: Sickle Cell Disease
SOMREC: School of Medicine Research Ethics Committee
UK: United Kingdom
USA: United States of America
VOC: Vaso-Occlusive Crisis
WHO: World Health Organization
